# Pharmacological interventions for Problematic Usage of the Internet (PUI): A narrative review of current progress and future directions

**DOI:** 10.1016/j.cobeha.2022.101158

**Published:** 2022-06-11

**Authors:** Jeremy E. Solly, Jon E. Grant, Samuel R. Chamberlain

**Affiliations:** a Cambridgeshire and Peterborough NHS Foundation Trust, Cambridge, UK; Department of Psychiatry, University of Cambridge, UK; b University of Chicago, Department of Psychiatry and Behavioral Neuroscience, Chicago, IL, USA; c Department of Psychiatry, Faculty of Medicine, University of Southampton, UK; Southern Health NHS Foundation Trust, Southampton, UK

## Abstract

Problematic Usage of the Internet (PUI) represents a spectrum of excessive online behaviors and is linked to reduced quality of life and high rates of psychiatric comorbidity, with growing demand for effective treatments. This paper provides a narrative review of pharmacological studies for PUI conducted to date. Most pharmacological treatment trials have focused on bupropion and escitalopram or involved samples with common comorbidities and used current treatments for the relevant comorbid disorders. Overall, there remains a dearth of high-quality evidence, with the current literature lacking control groups, large sample sizes, validated outcome measures, longer term treatment and follow-up periods. The literature cannot at this stage determine evidence-based pharmacological treatments for PUI.

## 1 Introduction

The Internet is a fundamental aspect of modern life but also represents a medium that can facilitate problematic behaviors for some individuals [1,2]. Problematic Usage of the Internet (PUI) represents a spectrum of excessive persisting online behaviors and can include gaming, buying/shopping, surfing, pornography use, gambling and social networking [1,3]. To date, gaming disorder and gambling disorder have been included in the International Classification of Diseases Version 11 (ICD-11) as behavioral addictions that may take place online or offline [4] and Internet Gaming Disorder (IGD) is a condition requiring further study in the Diagnostic and Statistical Manual Version 5 (DSM-5) [5]. Diagnostic criteria for PUI vary but include impaired control over problematic Internet-related activities, continued Internet use despite negative consequences and impaired functioning [1,4–6]. This paper does not concern whether these should constitute formal mental health disorders but addresses studies that have examined potential effects of medications on these types of excessive online activities.

PUI has been linked to reduced quality of life and high rates of psychiatric comorbidities [7–9] and is a growing public health concern, with clinical services established in some parts of the world to meet growing demand [10]. Although definitions of PUI and associated disorders vary, evidence from meta-analyses suggests that prevalence is high and rising over time [11,12]. Therefore, there is a need for evidence-based treatment strategies [1]. This narrative review collated current evidence for pharmacological therapies in PUI, aiming to summarize current progress and identify key questions for future research.

## 2 Method

PubMed and PsycINFO were searched up to 2^nd^ May 2022 using the following string, adapted from previous meta-analyses related to PUI [13,14]: (“drug” OR “medication*” OR “pharmacol*”) AND (“internet use” OR “internet addiction” OR “smartphone use” OR “smartphone addiction” OR “gaming addiction” OR “internet gaming disorder” OR “PIU” OR “PUI”). Terms related to “smartphone” addiction were used to ensure that the search included studies relevant to Internet use regardless of modality, while those related to “gaming” addiction were used to ensure IGD studies were included, as much of the available PUI literature focuses on this field.

Clinical trials involving the use of a pharmacological intervention in PUI were included in a narrative review. Reference lists of identified trials and relevant reviews were screened to identify further trials.

## 3 Pharmacological interventions to treat PUI as a primary disorder

Pharmacological trials evaluating treatment for PUI as a primary disorder have focused on the antidepressants bupropion and escitalopram.

Bupropion was first assessed as a candidate treatment for PUI with the hypothesis that it would reduce craving for Internet gaming [15]. Bupropion is a norepinephrine and dopamine reuptake inhibitor established to show efficacy in treating depression and has low rates of sexual side effects so is sometimes used for that reason [16]. It also shows efficacy in smoking cessation [17]. In the PUI trial, 12 male subjects (mean age 21.5 years) with Internet video game addiction and no comorbidities were treated with bupropion sustained release (SR) for six weeks, at a dose of 150 mg/day increasing to 300 mg/day [15]. One subject stopped treatment due to nausea. Following treatment, the authors found significant decreases in craving for the video game, total game play time and Young Internet Addiction Scale [18] (YIAS) scores. However, there was no control group. Given the small sample size and lack of a control condition, it is hard to know if the changes in symptoms were related to the medication or not.

A subsequent study based at the same research center investigated the effect of bupropion SR in 19 male subjects with IGD (mean age 25.3 years) and 18 male subjects with Internet-based Gambling Disorder (IbGD; mean age 25.0 years) and no comorbidities [19]. Bupropion SR was started at 150 mg/day, increased to 300 mg/day and continued for 12 weeks. Three subjects from each group dropped out. Significant decreases were noted in YIAS [18] and Yale-Brown Obsessive Compulsive Scale for pathological gambling [20] scores in the IGD and IbGD groups, respectively. Although the groups were compared to each other, there was no control condition.

Treatment of PUI with escitalopram was first suggested in a case report [21] and was subsequently evaluated in a trial [22]. Escitalopram is a selective serotonin reuptake inhibitor (SSRI) with established efficacy in treating depression [23]. 19 subjects (7 female, mean age 38.5 years) with so-termed impulsive-compulsive Internet usage disorder (involving various online activities, including email, pornography, gambling, searching or dating) were recruited, of which 12 had a comorbidity, mainly depression and social anxiety disorder [22]. Escitalopram was started at 10 mg/day and increased to 20 mg/day. There was an initial 10-week open-label phase, followed by a nine-week double-blind phase with subjects receiving escitalopram or placebo. Reported escitalopram side effects included drowsiness, nausea, fatigue and sexual side effects. Two subjects dropped out during the open-label phase and three dropped out during the double-blind phase (two in the escitalopram group and one in the placebo group), none due to side effects. After the open-label phase, weekly hours of Internet use and Clinical Global Impressions-Improvement scale [24] scores had significantly decreased, with 11 out of 17 patients considered to be responders. All 17 patients completing the open-label phase were randomized to escitalopram or placebo for nine further weeks. 14 completed this phase, with seven patients in each group. There were no significant differences between the escitalopram and placebo groups following the double-blind phase; however, this sample size is likely too small to make robust conclusions.

Bupropion and escitalopram have been compared for treating IGD in one open-label trial [25]. 119 male subjects were recruited from three research centers, including the center at which the two above studies of bupropion were based [15,19] and were randomly assigned to bupropion SR (at the same doses as previous studies), escitalopram (at the same doses as the previous study [22]) or observation (no medication) for six weeks. Mean ages of the three groups were 20.0, 19.8 and 19.6 years, respectively. YIAS [18] and Clinical Global Impressions-Severity [24] scores decreased in both treatment groups compared to the observation group, with greater decreases in the bupropion group. However, there was no placebo control and no data on side effects or dropout rates were reported.

## 4 Pharmacological interventions for PUI comorbid with another disorder

Epidemiological data suggest associations between PUI and a range of mental disorders, including major depressive disorder (MDD), substance use, attention-deficit/hyperactivity disorder (ADHD) and anxiety [9,26–28] and so treatment strategies accounting for these comorbidities are needed.

### 4.1 Major depressive disorder

Bupropion was evaluated in a placebo-controlled trial for treating MDD and excessive online game play, with game genres including role-playing, first-person shooters, real-time strategy and online gambling [29]. 57 male subjects received either bupropion SR or placebo and all took part in an education program. Mean ages were 21.2 and 19.1 years in the two groups, respectively. There was an eight-week double-blind treatment period and a four-week follow-up. Bupropion SR was started at 150 mg/day increasing to 300 mg/day, while placebo was started at one pill increasing to two. Four subjects receiving bupropion dropped out due to nausea and headache, while one subject receiving placebo dropped out due to nausea and two were excluded due to antidepressant treatment. At the end of the eight-week active treatment period, YIAS [18], Beck Depression Inventory (BDI) [30] scores and game playing time were all significantly reduced in the bupropion group compared to placebo. At follow-up four weeks after treatment was discontinued, YIAS scores and game playing time in the bupropion group remained stable, while BDI scores increased.

Another study based at the same research center compared a combination of bupropion and cognitive behavioral therapy (CBT) to bupropion alone in 72 male adolescents (age range 13-18 years) with MDD and problematic online game play [31]. All subjects received bupropion for eight weeks, at the same doses as previous studies, followed by a four-week follow-up. Six subjects discontinued bupropion due to side effects, including nausea, headache and palpitations. After eight weeks, the CBT group demonstrated reduced YIAS [18], BDI [30] scores and game playing time compared to bupropion alone, although outcomes improved in both groups. Beck Anxiety Inventory [32] scores increased in the group treated with bupropion alone but decreased with CBT. Outcomes remained the same at follow-up four weeks after completing treatment. There was no control group.

There has been one double-blind trial comparing bupropion to escitalopram in the treatment of 34 subjects with comorbid MDD and IGD, based at the same center as many of the previous studies [33]. Both groups (mean ages 22.9 and 23.9 years, respectively) were treated for 12 weeks, with medication doses the same as previous studies, and received an education intervention. Two subjects in the bupropion group dropped out due to side effects (nausea/diarrhea and palpitations), while one subject in the escitalopram group dropped out due to nausea/diarrhea and another was excluded due to mood switch. After 12 weeks, BDI [30] and YIAS [18] scores decreased in both groups, with no significant difference between them; however, the bupropion group demonstrated a significant decrease in attentional symptoms and impulsivity compared to escitalopram. There was no control arm, and the sex of subjects was not reported.

### 4.2 Attention-deficit/hyperactivity disorder

Methylphenidate is a psychostimulant medication that has established efficacy in treating ADHD [34]. A trial involving the same research center as the above studies evaluating bupropion assessed the effect of osmotic-release oral system (OROS) methylphenidate in 62 children (10 female, mean age 9.3 years) with ADHD who played Internet video games [35]. Problematic video game playing was not an inclusion criterion. Subjects received methylphenidate for eight weeks, starting at 18 mg/day, with the maintenance dose adjusted for each subject. Before study completion, nine subjects were discharged and 32 stopped treatment due to side effects (tic disorders, insomnia, poor appetite, abdominal discomfort/pain). After eight weeks, YIAS [18] scores and amount of time spent using the Internet had significantly reduced, alongside a reduction in ADHD symptoms. However, there was no control group, there was a high drop-out rate, and a majority of subjects (34) did not meet the typical YIAS cut-off for Internet addiction at baseline.

In a subsequent trial based at the same research center, atomoxetine and OROS methylphenidate were compared for treating 84 male adolescents with comorbid ADHD and IGD in a single-blind study for three months [36]. Atomoxetine is a noradrenaline reuptake inhibitor that is used as a treatment for ADHD [34,37]. Methylphenidate was started at 10 mg/day increasing to 40 mg/day, while atomoxetine was started at 10 mg/day increasing to 60 mg/day [36]. The mean ages in the two groups were 16.9 and 17.1 years, respectively. Following treatment, YIAS [18] scores improved similarly in both groups. ADHD symptoms improved more in the methylphenidate group. There was no control group and no information regarding side effects was reported.

### 4.3 Obsessive-compulsive disorder

Treatment of Internet addiction (IA) (another name for PUI) comorbid with obsessive-compulsive disorder (OCD) was evaluated in a naturalistic trial [38]. 27 OCD subjects without IA (mean age 29.4 years) and 11 OCD subjects with IA (mean age 21.2 years) received pharmacological treatment for OCD (clonazepam [benzodiazepine] tapered over three weeks, in addition to a SSRI or clomipramine [tricyclic]). Five subjects were lost to follow-up. Yale-Brown Obsessive Compulsive Scale (YBOCS) [39] and IAT [18] scores reduced in both groups, with YBOCS scores decreasing at similar rates in the two groups, while IAT scores decreased more in the IA group. At one year, two out of 11 IA subjects still met IA criteria. There was no control group.

### 4.4 Anxiety disorders

A treatment study used a combination of modified CBT (focusing on the relationship between anxiety and Internet use) and pharmacotherapy in 41 patients (28 female, mean age 28.9 years) with IA and an anxiety disorder (panic disorder or generalized anxiety disorder) [40]. Subjects were treated for 10 weeks with modified CBT and received a range of prescribed medications, including antidepressants, anxiolytics, psychostimulants and antipsychotics. Two subjects dropped out. Anxiety and IAT [18] scores significantly decreased following treatment. There was no control condition and the effects of different medications were not evaluated.

## 5 Other potential pharmacological interventions for PUI

Case reports suggest other medications that may merit investigation in treatment of PUI, such as a citalopram/quetiapine combination for treating uncontrollable “nonessential” Internet use [41] and naltrexone for reducing sexual urges in problematic usage of Internet pornography [42]. In a case series, paroxetine initially was associated with reduction of problematic pornography use but was later complicated by development of new offline compulsive sexual behaviors [43]. In fact, there have been numerous case reports of various medication treatments for compulsive sexual behaviors and therefore there is no biological reason to think that an online version of compulsive sexual behavior should or would respond differently from compulsive sexual behavior that does not involve technology. Finally, in an interview study of subjects with PUI, bipolar disorders were found to be the most common comorbid lifetime mood disorders and subjects reported that PUI had responded well to mood stabilizers in the past [44]. It should be noted that case reports are interesting but constitute a low grade of evidence.

Pharmacological interventions used in disorders related to PUI may also represent candidate treatments. For example, gambling disorder and gaming disorder are classified in the ICD-11 as disorders due to addictive behaviors that can occur either online or offline [4]. A variety of pharmacological interventions have been investigated for gambling, and treatments that have evidence of efficacy from at least one high quality randomized, double-blind, placebo-controlled study include certain opioid antagonists and the glutamatergic agent N-acetylcysteine (NAC) [45].

## 6 Conclusions

Despite growing interest there remains a lack of high-quality evidence regarding potential pharmacological interventions for PUI and candidate disorders falling under this umbrella term (e.g., IGD). Published trials focus on only a small range of pharmacotherapies and there appears to be only one study that is both double-blind and placebo-controlled [29] (Table 1). Overall, the available studies are insufficient in nature and scope to identify or refute evidence-based pharmacological treatments for PUI itself. However, they suggest that treating mental health disorders that have established evidence-based pharmacological treatments (such as comorbid depression or ADHD) should be a priority, including when those are present alongside PUI. The literature also highlights some medications that warrant clinical trial evaluation in future, such as bupropion, methylphenidate, naltrexone and NAC. These and potentially other pharmacotherapies warrant evaluation in suitably large, methodologically rigorous multi-site studies, but are contingent on the field both achieving consensus on how to define PUI and umbrella conditions reliably and identifying and validating outcome measures.

Common issues with study design in the current literature include a lack of control groups, small sample sizes, short periods of treatment, using non-validated outcome measures, not pre-specifying statistical plans, not accounting for drop-outs, lack of consideration of the many sources of potential bias, and lack of post-treatment follow-up [46,47]. In addition, there are important differences between studies in the definitions of PUI subtypes. There is also a need to include a wider range of populations, as many studies to date have included only male participants and most studies discussed here were conducted in a single setting.

In view of these limitations, meta-analysis of the currently available PUI pharmacological treatment literature is not meaningful or indicated at this stage. For example, meta-analysis of largely uncontrolled treatment studies would give a large effect size of benefit due to placebo effects and other non-specific effects, from which authors may erroneously conclude that the treatments have been shown to be effective, when this is not actually shown by available evidence. Establishing or refuting evidence-based pharmacological interventions for PUI, as noted, requires randomized, double-blind, placebo-controlled studies of appropriate size, duration, scientific quality, and methodological rigor. Similarly, it seems premature to combine pharmacological and psychological interventions in clinical trials for PUI when studies have not yet appropriately addressed pharmacological interventions alone.

Several limitations should be noted in relation to this paper. First, for pragmatic reasons we conducted our literature search using two main systems (PubMed and PsycInfo). As such, the findings may not be exhaustive in nature. Second, this review is narrative. A more detailed systematic review (with pre-registration), including using wider search strings (for example extending to other online activities more explicitly) may be useful to extend on these findings. At the same time, for the reasons outlined previously, meta-analysis would be invalid and likely to draw erroneous conclusions. There is also a lack of consensus on how to define many online activities clinically, which would make such a more detailed review challenging to conduct.

While more high-quality evidence regarding specific pharmacological treatments for PUI is awaited, a practical approach to treating patients presenting with PUI would be to screen for comorbidities and offer treatment for these according to established evidence-based guidelines, while monitoring PUI symptoms.

## 7 Future research directions

Research addressing the following points will contribute to the future evidence base for pharmacological interventions in PUI:

- Generating consensus definitions and diagnostic criteria for PUI and its subtypes to reduce heterogeneity between studies
- Identifying and validating clinical trial outcome measures appropriately
- Determining the clinical similarities and differences between related online and offline behaviors (for example, problematic gaming)
- Conducting scientifically rigorous and appropriately sized placebo-controlled trials in a wide range of populations and incorporating groups with different PUI subtypes and comorbidities. Trials should be pre-registered and should consider risk of bias and report methods clearly and completely (for example, by adhering to the CONSORT statement) [46–48].
- Evaluating pharmacological agents suggested by biological models and findings from other more studied settings (e.g., certain opioid or glutamatergic medications)

## Figures and Tables

**Table 1:** Summary of included studies

Study	Geographic area	PUI subtype(s)	Gender mix	Comorbid disorder(s)	Interventions	Control condition(s)	N	Treatment length	Follow-up length	Outcome measure(s)	Adverse events
Han et al., 2010 [15]	South Korea	Internet video game addiction	Male only	None	Bupropion	None	12	6 weeks	None	BDI, YIAS, game playing time, craving for playing the game (self-report with a visual analogue scale)	Nausea
Bae et al., 2018 [19]	South Korea	IGD and IbGD	Male only	None	Bupropion	None	37	12 weeks	None	BDI, BISBAS, ARS, YIAS, YBOCS, YBOCS-PG	None reported
Del’Osso et al., 2008 [22]	USA	IC-IUD	Mixed	Mainly MDD and social anxiety disorder	Escitalopram	9-week placebo-controlled discontinuation phase	19	19 weeks	None	Time spent on nonessential Internet use, CGI-I, BIS, IC-IUD-YBOCS	Drowsiness, nausea, fatigue, sexual side effects
Song et al., 2016 [25]	South Korea	IGD	Male only	None	Bupropion; Escitalopram	Observation (no medication) group	119	6 weeks	None	CGI-S, YIAS, BDI, ARS, BISBAS	No information
Han & Renshaw, 2012 [29]	South Korea	Online game addiction	Male only	MDD	Bupropion	Placebo	57	8 weeks	4 weeks	YIAS, BDI, CGI-S, game playing time	Nausea, headache
Kim et al., 2012 [31]	South Korea	Problematic online game play	Male only	MDD	Bupropion + CBT; Bupropion	None	72	8 weeks	4 weeks	YIAS, game playing time, BDI, BAI, Life-SS, School-PBS	Nausea, headache, palpitations
Nam et al., 2017 [33]	South Korea	IGD	Not reported	MDD	Bupropion + education; escitalopram + education	None	34	12 weeks	None	YIAS, BDI, ARS, BISBAS	Nausea, diarrhea, palpitations, mania
Han et al., 2009 [35]	South Korea	Internet video game players	Mixed	ADHD	Methylphenidate	None	62	8 weeks	None	YIAS, Internet usage time, ARS-PT, VCPT	Tic disorders, insomnia, poor appetite, abdominal discomfort/ pain
Park et al., 2016 [36]	South Korea	IGD	Male only	ADHD	Methylphenidate; atomoxetine	None	84	12 weeks	None	YIAS, CDI, ARS, BISBAS	No information
Bipeta et al., 2015 [38]	India	IA	Mixed	OCD	Clonazepam tapered over 3 weeks + SSRI/ clomipramine	None	38	1 year	None	YBOCS, IAT, YDQ	No information
Santos et al., 2016 [40]	Brazil	IA	Mixed	Panic disorder/ GAD	Modified CBT + various medications	None	39	10 weeks	None	IAT, HAM-A, HDRS, CGI	No information

ADHD, Attention-Deficit/Hyperactivity Disorder; ARS, ADHD Rating Scale; ARS-PT, ADHD Rating Scale, Parent and Teacher Version; BAI, Beck Anxiety Inventory; BDI, Beck Depression Inventory; BIS, Barratt Impulsiveness Scale; BISBAS, Behavioral Inhibition System and Behavioral Activation System scales; CBT, Cognitive Behavioral Therapy; CDI, Children’s Depression Inventory; CGI, Clinical Global Impressions scale; CGI-I, Clinical Global Impressions-Improvement scale; CGI-S, Clinical Global Impressions-Severity scale; GAD, Generalized Anxiety Disorder; HAM-A, Hamilton Anxiety Rating Scale; HDRS, Hamilton Depression Rating Scale; IA, Internet Addiction; IAT, Internet Addiction Test; IbGD, Internet-based Gambling Disorder; IC-IUD, Impulsive-Compulsive Internet Usage Disorder; IC-IUD-YBOCS, Impulsive-Compulsive Internet Usage Disorder version of the Yale-Brown Obsessive Compulsive Scale; IGD, Internet Gaming Disorder; Life-SS, Life Satisfaction Scale; MDD, Major Depressive Disorder; N, Number of subjects; OCD, Obsessive-Compulsive Disorder; School-PBS, School Problematic Behavior Scale; SSRI, Selective Serotonin Reuptake Inhibitor; VCPT, Visual Continuous Performance Test; YBOCS, Yale-Brown Obsessive Compulsive Scale; YBOCS-PG, Yale-Brown Obsessive Compulsive Scale for Pathological Gambling; YDQ, Young’s Diagnostic Questionnaire; YIAS, Young Internet Addiction Scale.

## Highlights

- Methodological issues are common in trials of pharmacological treatments for PUI
- Available clinical studies do not support any specific evidence-based treatments
- Existing literature draws attention to the value of treating comorbidities
- We highlight future directions so that evidence-based treatments can be identified
